# Supplementary material for: Machine learning for determining lateral flow device results for testing of SARS-CoV-2 infection in asymptomatic populations
Source: Cell Rep Med. 2022 Sep 27;3(10):100784. doi: 10.1016/j.xcrm.2022.100784 (PMC9513327; doi:10.1016/j.xcrm.2022.100784)
Supplement: Document S1. Table S1 [file mmc1.pdf]

Cell Reports Medicine, Volume *3*

Supplemental information

Machine learning for determining lateral flow device results for testing of SARS-CoV-2 infection in asymptomatic populations

The LFD AI Consortium

Supplementary Materials for Beggs et al

Supplementary table 1: Table of viral loads in reference specimens as determined by real time PCR of ORF1ab and N genes vs. supplied concentrations of PFU/ml. Related to Figure 2.

PFU/ml	Ct ORF1ab	Ct N
10000	17.9	21.7
1000	24.3	27.4
100	27.4	30.3
10	33.5	35.2
1	35.6	38.6

List of affiliations of secondary authors:

NHS Test & Trace: Robert Banathy, Mark Branigan, Paul Lewis-Borman, Nishali Patel, Lennard Lee, Tom Fowler

Durham University; Camila C. S. Caiado

Sensyne Plc: Anna Dijkstra, Piotr Chudzik, Paria Yousefi, Avelino Javer, Bram Van Meurs, Lionel Tarassenko, Benjamin Irving

University of Birmingham: Celina Whalley, Neeraj Lal, Helen Robbins, Elaine Leung, Andrew D Beggs
