# Supplementary material for: Machine learning for determining lateral flow device results for testing of SARS-CoV-2 infection in asymptomatic populations
Source: Cell Rep Med. 2022 Sep 27;3(10):100784. doi: 10.1016/j.xcrm.2022.100784 (PMC9513327; doi:10.1016/j.xcrm.2022.100784)
Supplement: Document S2. Article plus supplemental information [file mmc2.pdf]

Cell Reports
Medicine

Article

Machine learning for determining lateral flow device results for testing of SARS-CoV-2 infection in asymptomatic populations

Graphical abstract

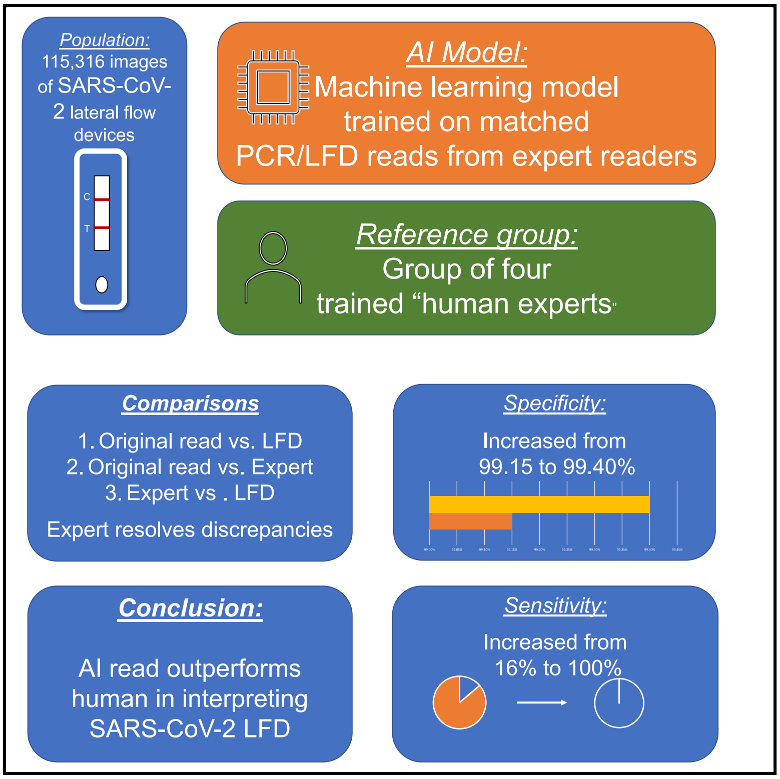

Authors

The LFD AI Consortium

Correspondence

a.beggs@bham.ac.uk

In brief

The AI LFD Consortium demonstrates that addition of a machine learning-based reader of lateral flow devices increases the accuracy of returned results for SARS-CoV-2 positivity.

Highlights

- Lateral flow devices are used as a rapid method of detecting SARS-CoV-2
- The accuracy of these devices is limited by the user
- This study shows an AI model increases the accuracy of returned results
- A greater than 25% increase in accuracy was seen

The LFD AI Consortium, 2022, Cell Reports Medicine *3*, 100784
October 18, 2022 © 2022 The Author(s).
https://doi.org/10.1016/j.xcrm.2022.100784

CellPress

Cell Reports Medicine

CellPress
OPEN ACCESS

Article

Machine learning for determining lateral flow device results for testing of SARS-CoV-2 infection in asymptomatic populations

The LFD AI Consortium[1,*]
[1]Lead contact (Andrew D. Beggs)
*Correspondence: a.beggs@bham.ac.uk (A.D.B.)

SUMMARY

Rapid antigen tests in the form of lateral flow devices (LFDs) allow testing of a large population for severe acute respiratory syndrome coronavirus 2 (SARS-CoV-2). To reduce the variability in device interpretation, we show the design and testing of an artifical intelligence (AI) algorithm based on machine learning. The machine learning (ML) algorithm is trained on a combination of artificially hybridized LFDs and LFD data linked to quantitative real-time PCR results. Participants are recruited from assisted test sites (ATSs) and health care workers undertaking self-testing, and images are analyzed using the ML algorithm. A panel of trained clinicians is used to resolve discrepancies. In total, 115,316 images are returned. In the ATS substudy, sensitivity increased from 92.08% to 97.6% and specificity from 99.85% to 99.99%. In the self-read substudy, sensitivity increased from 16.00% to 100% and specificity from 99.15% to 99.40%. An ML-based classifier of LFD results outperforms human reads in assisted testing sites and self-reading.

INTRODUCTION

Severe acute respiratory syndrome coronavirus 2 (SARS-CoV-2) is a betacoronavirus responsible for coronavirus disease 2019 (COVID-19).[1] Assessment of the extent of infection has largely been based on real-time polymerase chain reaction (PCR), which identifies the virus in those with infection. However, in the UK, antigen testing is predominantly performed in those presenting with symptoms (which may represent a small fraction of those infected with SARS-CoV-2) or as part of an asymptomatic testing program.

Recent evidence has demonstrated that 17%–33% of individuals are asymptomatic, and around 49% of individuals initially defined as asymptomatic eventually develop symptoms.[2] There continues to be uncertainty regarding the extent to which asymptomatic individuals transmit SARS-CoV-2. Viral culture studies suggest that people with SARS-CoV-2 can become infectious 1–2 days before onset of symptoms and continue to be infectious up to 7 days thereafter and that viable virus is relatively short lived.[3]

The U K has adopted a multifaceted testing strategy to support diagnostic, testing, and surveillance strategies.[4–7] Asymptomatic testing to support entry testing has been recommended as an effective strategy to suppress transmission but requires rapid results.[8] Frequent and serial testing of large fractions of the population has been highlighted as a powerful mechanism for outbreak suppression.[8]

Lateral flow devices (LFDs) are relatively easy-to-use rapid tests that can be performed in home settings by trained or lay users or near the point of care without the need for laboratory infrastructure or expensive equipment. There are two types of SARS-CoV-2 LFDs: virus antigen(s) tests (which were used in this study), which test for protein produced by the virus, and antibody tests (requiring blood sampling and possibly requiring spinning down and aliquoting of plasma when they are not compatible with whole blood), which detect one or more types of antibodies produced by the host immune response against the virus.

Although rapid antigen tests have lower analytical sensitivity (i.e., require greater amounts of virus material to turn positive) than qPCR-based tests, their ability to detect infectious individuals with culturable virus is as high as for qPCR.[9] Results from analyses conducted in a city-wide testing program in late 2020, where LFT results using a device from Innova were compared with comparable cycle threshold (Ct) levels from a follow-up PCR test showed that a decrease in Ct level (i.e., an increase in viral load) corresponded with a positive LFD.[4] Compared with quantitative real-time PCR, the sensitivity of the test has been found to be between 48.9% and 78.8% and the specificity in excess of 99.9% in mass testing.[10] Several other LFDs from different manufacturers are in the pipeline to be publicly released.[10] The majority of LFDs are designed to be interpreted visually, and this interpretation involves looking at two possible horizontal lines on the device, representing the control and the test lines. Presence of the control line assures the user that the test has been performed correctly, and the test line (if present) indicates that the presence of SARS-CoV-2 antigen has been detected.

A potential issue is that human eye acuity varies from person to person and is subject to one's individual characteristics, such as sharpness of the retinal image, health and function of the retina, and sensitivity of the interpretative faculty of the brain. In moving

This is an open access article under the CC BY license (http://creativecommons.org/licenses/by/4.0/).

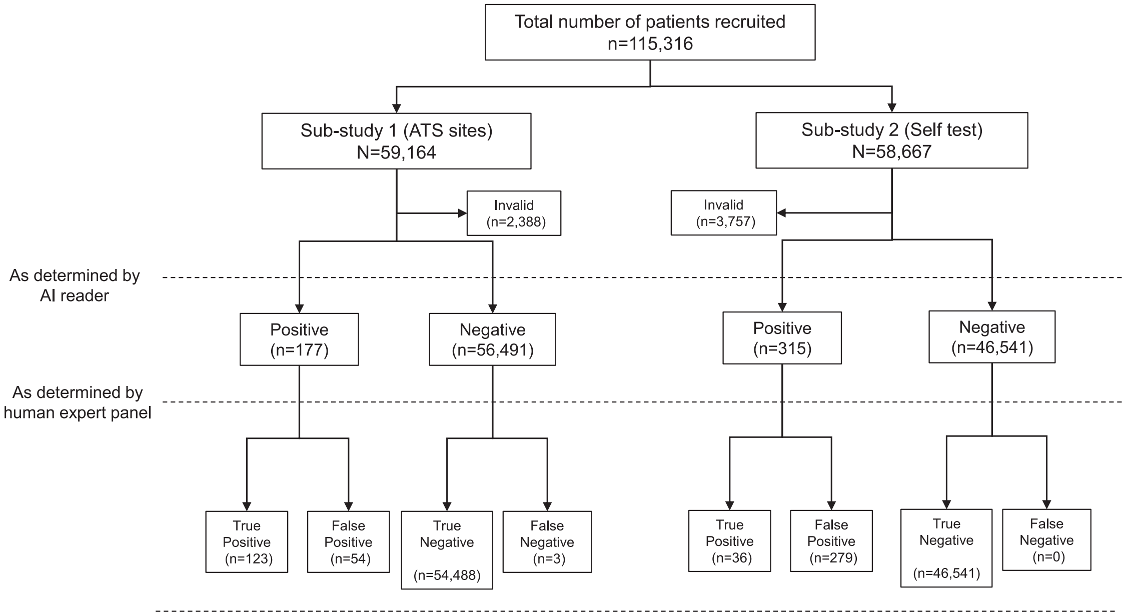

Figure 1. Flowchart of participants through the study
Voids are not shown.

to mass testing using LFDs, there is a potential concern that has been identified in that there is a risk of users interpreting the test incorrectly,[11] particularly when the test lines are extremely faint because of low viral loads. As a result, positive individuals would be missed during testing, and there is a risk that these individuals would contribute to a chain of transmission. As use cases expand, these may also begin to encompass the young (especially children under 11, who should be supervised when using the device) or very elderly individuals, who may find it challenging to properly interpret the results. We also expect such a system to enable support of members of the public who are not confident in using these devices. To help mitigate this risk, we developed a machine learning model to interpret user-taken photos of LFDs from the United Kingdom asymptomatic testing program. We hypothesized that the AI model would demonstrate accuracy at least equivalent to trained expert interpretation at assisted testing sites (ATSs).

RESULTS

Intra-reviewer accuracy

The three reviewers agreed with each other at least 94% of the time (pairwise agreement). Reviewer 1 and reviewer 2 agreed 94.6% of the time, reviewer 1 and reviewer 3 agreed 96.4% of the time, and reviewer 2 and reviewer 3 agreed 97.2% of the time. Adjusting for random agreement, the three pairs have estimated Cohen's kappa values of 0.83, 0.89, and 0.92, respectively, indicating very strong agreement.

When examining the samples reviewer 4 was asked to review, agreement between reviewer 2 and reviewer 3 remained moderate (kappa 0.48), but reviewer 1 agreement with reviewer 2, and reviewer 3 was marginal (below 0.05). Reviewer 4 was in slight agreement with reviewer 1 (0.11), fair agreement with reviewer 2 (0.28), and moderate agreement with reviewer 3 (0.59). These cases represented images where there was considerable diagnostic uncertainty because of the test (T) lines being at the edge of detectability, and this may explain the poor correlations when reviewer 4 was introduced. Given this, the levels of agreement were consistent and reasonable, and so we concluded that we should continue to use them for review and bring in reviewer 4 when needed to resolve a conflict.

Substudy 1

There were 59,164 images submitted (Figure 1) via the ATSs and used in this analysis. There were 2,388 images that were labeled as invalid and rejected; had the algorithm been in production mode, these users would have been asked to take another photo to improve image quality, leaving 56,776 valid images.

Taking the ATS user label as reference (i.e., the trained operator's classification of the test), the estimated specificity and sensitivity were 99.85% and 92.08%, respectively (Table 1). The void accuracy was 56.67%, and the overall accuracy of the algorithm was estimated at 99.67%. The multiclass area under the receiver operating characteristic curve (AUROC) value was 0.98. A representative selection of imaged LFDs are shown in Figure 2.

The next step was to re-evaluate the "ground truth" whereby discrepancies identified between the AI algorithm and the ATS operators were arbitrated by the reviewer clinicians to create a "most trusted result" as outlined above. There were 13 cases where absolute majority or agreement with the fourth reviewer was reached. Two of those cases had an ATS label of negative; two reviewers labeled them as positive, and so did reviewer 4. These cases were deemed to be positive. There were 10 cases

Table 1. Confusion matrix for cases that passed QC, showing ATS and MagnifEye labels (substudy 1)

		MagnifEye label		
		Positive	Negative	Void
ATS label (Original not clinically assessed)	Positive	93	8	0
	Negative	85	56,463	67
	Void	1	25	34
		95% confidence interval (CI)		
Sensitivity	92.08%	84.99–96.52%		
Specificity	99.85%	99.81%–99.88%		
Positive predictive value	52.25%	46.75–57.69%		
Negative predictive value	99.99%	99.97–99.99%		
Void accuracy				
Accuracy	99.84%	99.80–99.87%		

read as negative by the ATS operatives; the AI algorithm's classification was positive, two reviewers labeled the test results as positive, but one reviewer labeled those as negative. After further inspection, a faint line was visible in most of the images, and these were truly edge cases; however, the two reviewers that labeled these cases as positive were the most consistent throughout the process, so we chose to take their majority (2 of 3) view and label these cases as positive. One case was classified as negative by the AI algorithm, positive by ATS, positive by two reviewers, unable to be classified by one reviewer, and void by the fourth reviewer. The test was ultimately classified as positive. After further inspection, this was a case where the full test could not be seen in the image, and it did not meet quality control; this is what led reviewer 4 to classify it as void. Because it did not fail the automated quality control, the case was kept.

There were 3 cases the AI algorithm read as negative and the human reviewer vote was positive, with the ATS label also positive. Two reviewers labeled them as positive, one reviewer labeled them as negative, and reviewer 4 as positive. These cases were taken to be positive. There was a fourth case the AI algorithm read as negative, the ATS as positive, one reviewer was unable to classify, one returned positive, one returned negative, and the fourth returned void. Because the "ground truth" could not be established, the case was removed from the overall analysis.

The reviewers checked all positive and void cases where the ATS and the AI algorithm agreed, and the reviewers returned an absolute majority for all. They also checked a sample of negative cases where the ATS and the AI algorithm agreed (n = 568); full majority was returned in 566 cases and a simple majority in the remaining 2 cases. We took the remaining cases where there was agreement between the algorithm and ATS on negatives to be correctly classified.

After resolution of discrepancies (Table 2), comparing the ground truth with the ATS labels, the estimated specificity was greater than 99.9%, and sensitivity was 97.6%. Void detection accuracy was 83.67% (requiring further improvement), and further analysis needs to be undertaken. Overall accuracy was 99.78%. The bootstrapped 95% confidence interval for the sensitivity was 94.44%–100%, which meets the criteria established for this study. The multiclass AUROC was close to 1.0. The number of true positives identified increased from 93 (identified by the trained ATS operators) to 123 (identified by the AI algorithm and confirmed by clinical reviewers).

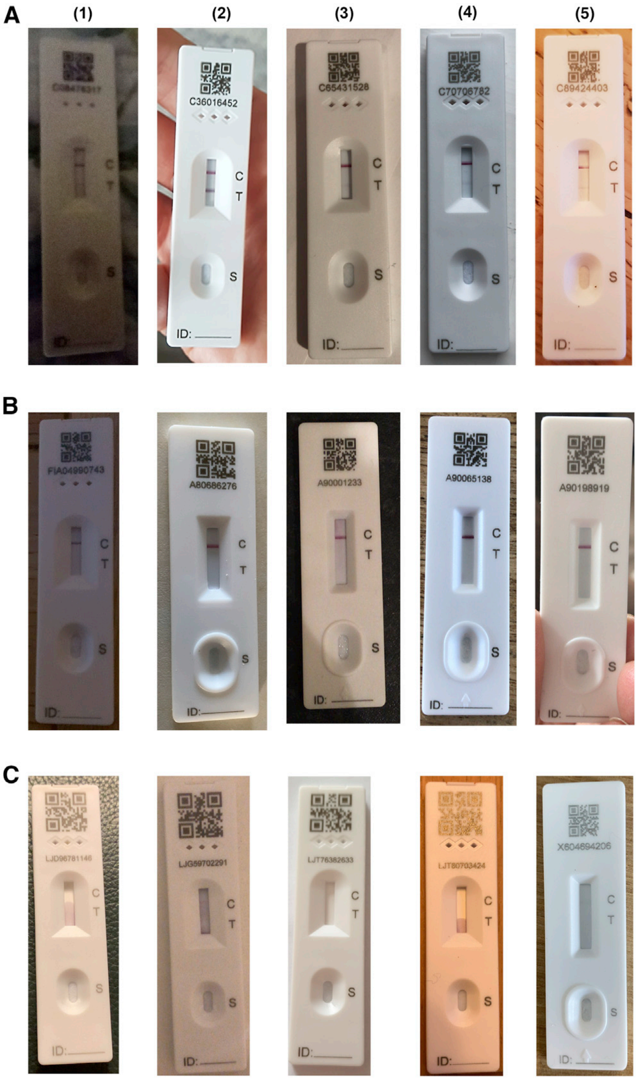

Figure 2. Representative LFDs for positives, negatives, and Voids
(A–C) Representative LFDs for (A) positives, (B) negatives, and (C) voids. For each row, columns (1–5) represent different lighting and angulated and focus conditions to represent a selection of real-world samples.

The AI algorithm's ability to correctly detect voids needs improvement because there are still 58 cases labeled as void by the algorithm that were labeled as negative by the reviewers. After closer inspection, we observed that the majority of cases were negative tests that were upside down. Mitigation will be possible by improving the quality control stages and the instructions to the user. We suggest that the next step in quality control development would be to include better rotation rejection or the ability to rotate the device more than 30°. We assessed the calibration of the model by use of the Brier score. This was low at 0.018, indicating good model calibration (closer to 0 represents

Table 2. Confusion matrix showing the "ground truth" and MagnifEye labels (substudy 1)

		MagnifEye label		
		Positive	Negative	Void
Ground truth (clinically assessed)	Positive	123	3	0
	Negative	54	56,488	58
	void	2	6	41

		95% CI
Sensitivity	97.6%	93.20%–99.51%
Specificity	99.95%	99.93%–99.98%
Positive predictive value	69.5%	63.53–74.86%
Negative predictive value	99.9%	99.98–100.0%
Void accuracy	83.7%	
Accuracy	99.7%	

The table has one less case where the ground truth could not be established and has been removed.

Table 3. Confusion matrix showing the user-submitted result and the AI read (substudy 2)

		MagnifEye label		
		Positive	Negative	Void
User read	positive	2	12	0
	negative	265	32,736	248
	void	0	8	8

		95% CI
Sensitivity	14.29%	1.78%–42.81%
Specificity	99.20%	99.09%–99.29%
Positive predictive value	0.75%	0.09%–2.68%
Negative predictive value	99.96%	99.94–99.98%
Void accuracy		
Accuracy		

a better calibration of the model.). When utilizing the quality control measures implemented in the MagnifEye software, the observed Brier score reduced to 0.013.

Substudy 2

We received 50,999 valid cases from National Health Service (NHS) users (Figure 1), with 3,757 submissions deemed invalid in cases that were labeled as positive or void by the user or the AI algorithm. These and all other cases where there was disagreement between the user and the AI algorithm, as well as a sample of negatives (n = 464), were reviewed.

The number of positive cases was too small for an accurate estimate, but specificity and sensitivity were high (greater than 99%). We investigated the 279 cases misidentified as positive by the AI algorithm, and the majority had potential quality-related issues, including shadows where the positive line would have been or unsuitable backgrounds. At least 5 devices seemed to be damaged by the user (e.g., leakage) or contaminated. The number of identified true positives increased from 4 (Table 3) to 36 (Table 4) when moving from the user read to the AI algorithm (Table 5).

We also investigated the returned confidence levels and anomaly scores for these cases and voids. We found that most were borderline, and, had rejection thresholds been more stringent for positive cases, the user would have been asked to retake a more appropriate photo in a production setting. There is a clear case for further improvements to the front end and instructions passed to the user to help reduce the number of failures because of incorrectly oriented tests (e.g., upside down), shadows, and low-resolution images.

Missing data

Only one image was removed from substudy 1, where the "ground truth" could not be established even after including a fourth reviewer, the chief investigator. Its inclusion as a false negative or a false positive would not have substantially affected the estimates of sensitivity and specificity.

As already described, the MagnifEye software tool includes automated quality control measures. In substudy 1, 59,164 images were submitted, and 2,388 were classified as invalid, most as a consequence of low-quality or rotated images. The remaining 56,776 images were considered valid and were then classified as positive, negative, or void.

In substudy 2, 51,001 images were submitted. Of those, 3,757 were classified as invalid. Two cases were incomplete, and the user reads were not recorded, and/or the user journeys were not completed appropriately; these two cases were excluded. It was not possible to establish "ground truth" for one case because all reviewers reported different responses, and the case was also excluded from the analysis.

DISCUSSION

We carried out one of the world's first diagnostic accuracy studies of a machine learning-based image reader embedded in a national health service reporting platform using a web interface to read the results of LFD tests taken by untrained participants in an asymptomatic population. A recent publication has utilized AI software in a dedicated reader with trained operatives to interpret HIV LFDs, demonstrating reasonable sensitivity and specificity,[12] differing from this study because the MagnifEye algorithm was deployed "into the wild" with a non-trained user base. We demonstrated that the novel AI algorithm, implemented in the MagnifEye software tool, performs well using Innova LFDs, with high sensitivity (97.9%) and specificity (>99.9%) in the ATS setting, and, when used in a self-test setting, sensitivity and specificity exceeded 99%. We hypothesize that the very substantial increase in testing accuracy is a direct result of the AI algorithm correctly classifying LFD results better than their human reads, especially for marginal cases with faint T lines. More importantly, at the low population prevalence seen in this asymptomatic testing study, the observed positive and negative predictive values produced by the AI algorithm are high enough to be utilized for routine use. The positive predictive value of greater than 65% in each cohort seems at first examination to be lower than what would be sensible for a population diagnostic test; however, with a low population prevalence of positivity because of use in an asymptomatic population where this would always be the case, the positive

Table 4. Confusion matrix showing the "ground truth" result and the AI read (substudy 2)

		MagnifEye label		
		Positive	Negative	Void
Ground truth (clinically assessed)	positive	30	0	1
	negative	236	32,750	222
	void	0	5	10

		95% CI
Sensitivity	100%	90.26%–100%
Specificity	99.28%	99.19%–99.37%223
Positive predictive value	11.28%	7.74–15.71%
Negative predictive value	100%	
Void accuracy	66.67%	
Accuracy	98.60%	98.47%–98.73%

Table 5. Confusion matrix showing the "ground truth" result and the user read (substudy 2, excluding invalid images)

		User read		
		Positive	Negative	Void
Ground truth (clinically assessed)	positive	2	12	0
	negative	29	33,193	2
	void	0	3	13

		95% CI
Sensitivity	6.45%	0.79%–21.42%
Specificity	99.96%	99.94%–99.98%
Positive predictive value	14.29%	0.18%–42.81%
Negative predictive value	99.91%	99.87%–99.94%
Void accuracy	81.25%	54.35%–95.95%
Accuracy	99.86%	99.82%–99.90%

predictive value (PPV) of this test is comparable with other population studies on PCR. However, with a large population, a disadvantage of this system is that, even with an extremely low false positive rate, additional tests will be detected as being incorrectly positive given the size of the population studied; however, the false positive rate observed is still less than the human "lay" read. Diagnostic test development is a fine balance between overcalling and undercalling, and because of the serious consequences of missing a case of SARS-CoV-2, we made the decision to set the parameters of the algorithm so that positivity is overcalled. This is also inflated because of the low population prevalence of SARS-CoV-2 in the study, a direct reflection of the fact that, in all asymptomatic diagnostic studies of SARS-CoV-2 to date, the prevalence has been between 0.1% and 1%, which means that the false positive rate is artificially high. In a higher-prevalence population, this false positive rate would be significantly lower.

We found that the system correctly identified positive and negative results that had been visually classified by users as being the opposite, meaning that the AI algorithm could provide the ground truth with accuracy beyond a human reader and therefore could reduce the documented false positive and false negative rates seen with the LFD tests, which are in part due to user read error. This was validated by the external validation panel of four clinicians we utilized for this program. Because the AI algorithm performs identically on an identical image, its consistency is another advantage. A further advantage is that the processing time of the MagnifEye software is below 2 s (typically 0.5 s), even at peak load (500 tests analyzed per second), meaning a result can be returned rapidly to the end user. The MagnifEye software successfully detected low viral loads that human readers failed to mark as positive. MagnifEye has the potential to assist users who are uncertain about reading LFDs in the testing journey by returning to them an accurate and reproducible classification result. We made a decision to use an expert panel of readers as ground truth rather than a molecular ground truth such as quantitative real-time PCR because the dynamic range of detection of these tests is different, and our study set out to measure the accuracy of detecting bands on the test, not to specifically detect the presence of SARS-CoV-2 infection accurately.

A weakness of the AI algorithm is the need for a reasonable-quality image to be taken for it to read the result accurately. In our study, a reasonable percentage (4.5%) of the images were rejected for not meeting the specifications required to pass quality control, meaning the user would have needed to take the image again. This stringent quality control (QC) has the advantage of ensuring that only good-quality images are taken but may lead to user fatigue or non-compliance with the algorithm if they find it difficult to take good-quality images. Anecdotally, we found a very small amount of totally unsuitable images (extremely small test in image, bright reflections or severe shadows, non-white light, blurry or very dark images), which suggested that more training or visual aids would be of benefit to end users to ensure consistency in the image acquisition process. Another potential issue is access to this technology in disadvantaged groups (elderly, homeless, refugees) or those without a smartphone. A particularly difficult group to reach with LFDs are users from socioeconomic groups CDE,[4] however, the Office of National Statistics has demonstrated that these groups have smartphone access with groups AB, suggesting that this algorithm could potentially improve accessibility for this hard-to-reach group.[13]

We carried out this pilot study in two scenarios; the first in assisted test centers, where a standardized protocol was used to take the image, and the second in self-test settings, where healthcare workers took the images themselves. We found no difference in rejection rates between the two groups, suggesting that each was equally proficient at taking images, and we also found high levels of sensitivity and specificity in both scenarios. The AI algorithm in the MagnifEye software tool may be generalizable to other use cases, such as school testing and testing for members of the general public, but this pilot study did not specifically assess these groups, and so a period of testing may be required for these groups.

The MagnifEye automated LFD reader, in this diagnostic test study, has been demonstrated to work at a level consistent with accurate performance in a live clinical environment, processing millions of images a day, allowing rapid, consistent, and accurate feedback of test results to the end user and recording by central government. MagnifEye allows

standardization of test reporting, increasing confidence in LFD testing, reducing user error, and increasing the overall accuracy of an AI LFD program. Although the impact of SARS-CoV-2 has been markedly reduced through vaccination, many parts of the world have limited access to this. Evolution of the virus has led to subsequent waves and the need for rapid deployment of measures of infection control, in which this reader can play a useful role.

Limitations of the study

The major limitation of this study is that it only considers interpretation of LFDs by the human reader. The accuracy of these devices in detection of SARS-CoV-2 has other important determinants, including the viral load, type of device used, and competency of the user in performing the test according to manufacturer's instructions.

CONSORTIA

The members of the LFD AI Consortium are Andrew D. Beggs, Camila C.S. Caiado, Mark Branigan, Paul Lewis-Borman, Nishali Patel, Tom Fowler, Anna Dijkstra, Piotr Chudzik, Paria Yousefi, Avelino Javer, Bram Van Meurs, Lionel Tarassenko, Benjamin Irving, Celina Whalley, Neeraj Lal, Helen Robbins, Elaine Leung, Lennard Lee, and Robert Banathy.

STAR★METHODS

Detailed methods are provided in the online version of this paper and include the following:

- KEY RESOURCES TABLE
- RESOURCE AVAILABILITY
  - Lead contact
  - Materials availability
  - Data and code availability
- EXPERIMENTAL MODEL AND SUBJECT DETAILS
  - Algorithm details
  - Participants
- METHOD DETAILS
  - Study design
  - Image capture
  - Resolution of discrepancies & intra-observer reliability
- QUANTIFICATION AND STATISTICAL ANALYSIS
  - Sample size
  - Calculation of accuracy
- ADDITIONAL RESOURCES
  - Study registration

SUPPLEMENTAL INFORMATION

Supplemental information can be found online at https://doi.org/10.1016/j.xcrm.2022.100784.

ACKNOWLEDGMENTS

The study was funded by NHS Test and Trace. A.D.B. is funded by an CRUK Advanced Clinician Scientist Award (C31641/A23923). The funder had input into design and conduct but not reporting of the study.

AUTHOR CONTRIBUTIONS

Study conception and design A.D.B. and the LFD AI Consortium; algorithmic design and implementation, the LFD AI Consortium; laboratory work: A.D.B. and the LFD AI Consortium; statistical analysis, A.D.B. and the LFD AI Consortium; writing of the manuscript, A.D.B. and the LFD AI Consortium. All authors had access to the data presented in the manuscript.

DECLARATION OF INTERESTS

The authors declare no competing interests.

INCLUSION AND DIVERSITY

We worked to ensure gender balance in the recruitment of human subjects. We worked to ensure ethnic or other types of diversity in the recruitment of human subjects. We worked to ensure that the study questionnaires were prepared in an inclusive way. One or more of the authors of this paper self-identifies as an underrepresented ethnic minority in science. One or more of the authors of this paper self-identifies as living with a disability.

Received: April 24, 2022
Revised: June 25, 2022
Accepted: September 22, 2022
Published: October 18, 2022

REFERENCES

1. Zhang, J.J., Dong, X., Cao, Y.Y., Yuan, Y.D., Yang, Y.B., Yan, Y.Q., Akdis, C.A., and Gao, Y.D. (2020). Clinical characteristics of 140 patients infected with SARS-CoV-2 in Wuhan, China. Allergy *75*, 1730–1741. https://doi.org/10.1111/all.14238.
2. Cevik, M., Tate, M., Lloyd, O., Maraolo, A.E., Schafers, J., and Ho, A. (2021). SARS-CoV-2, SARS-CoV, and MERS-CoV viral load dynamics, duration of viral shedding, and infectiousness: a systematic review and meta-analysis. Lancet. Microbe *2*, e13–e22. https://doi.org/10.1016/S2666-5247(20)30172-5.
3. La Scola, B., Le Bideau, M., Andreani, J., Hoang, V.T., Grimaldier, C., Colson, P., Gautret, P., and Raoult, D. (2020). Viral RNA load as determined by cell culture as a management tool for discharge of SARS-CoV-2 patients from infectious disease wards. Eur. J. Clin. Microbiol. Infect. Dis. *39*, 1059–1061. https://doi.org/10.1007/s10096-020-03913-9.
4. García-Fiñana, M., Hughes, D.M., Cheyne, C.P., Burnside, G., Stockbridge, M., Fowler, T.A., Fowler, V.L., Wilcox, M.H., Semple, M., and Buchan, I. (2021). Performance of the Innova SARS-CoV-2 Antigen Rapid Lateral Flow Test in the Liverpool Asymptomatic Testing Pilot (SSRN). https://doi.org/10.2139/ssrn.3798558.
5. Ptasinska, A., Whalley, C., Bosworth, A., Poxon, C., Bryer, C., Machin, N., Grippon, S., Wise, E.L., Armson, B., Howson, E.L.A., et al. (2021). Diagnostic accuracy of loop mediated isothermal amplification coupled to nanopore sequencing (LamPORE) for the detection of SARS-CoV-2 infection at scale in symptomatic and asymptomatic populations. Clin. Microbiol. Infect. *27*, 1348–1348.e7. https://doi.org/10.1016/j.cmi.2021.04.008.
6. Richter, A., Plant, T., Kidd, M., Bosworth, A., Mayhew, M., Megram, O., Ashworth, F., Crawford, L., White, T., Moles-Garcia, E., et al. (2020). How to establish an academic SARS-CoV-2 testing laboratory. Nat. Microbiol. *5*, 1452–1454. https://doi.org/10.1038/s41564-020-00818-3.
7. Shields, A., Faustini, S.E., Perez-Toledo, M., Jossi, S., Aldera, E., Allen, J.D., Al-Taei, S., Backhouse, C., Bosworth, A., Dunbar, L.A., et al. (2020). SARS-CoV-2 seroprevalence and asymptomatic viral carriage in healthcare workers: a cross-sectional study. Thorax *75*, 1089–1094. https://doi.org/10.1136/thoraxjnl-2020-215414.
8. Pavelka, M., Van-Zandvoort, K., Abbott, S., Sherratt, K., Majdan, M., Krajčí, M., Flasche, S., Flasche, S., and Funk, S.CMMID COVID-19 working group; Inštitút Zdravotných Analýz (2021). The impact of population-wide rapid

antigen testing on SARS-CoV-2 prevalence in Slovakia. Science *372*, 635–641. https://doi.org/10.1126/science.abf9648.

9. Lee, L.Y.W., Rozmanowski, S., Pang, M., Charlett, A., Anderson, C., Hughes, G.J., Barnard, M., Peto, L., Vipond, R., Sienkiewicz, A., et al. (2022). SARS-CoV-2 infectivity by viral load, S gene variants and demographic factors and the utility of lateral flow devices to prevent transmission. Clin. Infect. Dis. *74*, 407–415. https://doi.org/10.1093/cid/ciab421.
10. Peto, T. (2021). COVID-19: rapid Antigen detection for SARS-CoV-2 by lateral flow assay: a national systematic evaluation for mass-testing. Preprint at medRxiv. https://doi.org/10.1101/2021.01.13.21249563.
11. Mattioli, I.A., Hassan, A., Oliveira, O.N., Jr., and Crespilho, F.N. (2020). On the challenges for the diagnosis of SARS-CoV-2 based on a review of current methodologies. ACS Sens. *5*, 3655–3677. https://doi.org/10.1021/acssensors.0c01382.
12. Turbé, V., Herbst, C., Mngomezulu, T., Meshkinfamfard, S., Dlamini, N., Mhlongo, T., Smit, T., Cherepanova, V., Shimada, K., Budd, J., et al. (2021). Deep learning of HIV field-based rapid tests. Nat. Med. *27*, 1165–1170. https://doi.org/10.1038/s41591-021-01384-9.
13. Statista. (2021). Mobile Phone Usage by Socioeconomic Group. https://www.statista.com/statistics/300421/smartphone-usage-in-the-uk-by-socio-economic-group/.
14. Bowden, J., and Trippa, L. (2017). Unbiased estimation for response adaptive clinical trials. Stat. Methods Med. Res. *26*, 2376–2388. https://doi.org/10.1177/0962280215597716.
15. Emerson, S.S., and Fleming, T.R. (1990). Parameter estimation following group sequential hypothesis testing. Biometrika *77*, 875–892. https://doi.org/10.2307/2337110.

STAR★METHODS

KEY RESOURCES TABLE

REAGENT or RESOURCE	SOURCE	IDENTIFIER
Biological samples		
X-ray inactivated SARS-CoV-2 (England strain 1)	UK Health Security Agency, Porton Down, U.K.	https://www.gov.uk/government/organisations/uk-health-security-agency
Critical commercial assays		
Innova type A,B,C and D Lateral Flow Devices for detection of SARS-CoV-2	Innova Medical Group, Pasadena, California, U.SA.	https://innovamedgroup.com/innova-rapid-antigen-test/
Deposited data		
Algorithm training methodology	This paper	https://data.mendeley.com/datasets/hj9hmywh9c/1
Software and algorithms		
MagnifEYE algorithm	Sensyne Plc	https://www.sensynehealth.com/magnifeye This algorithm is not publicly available due to it being sold for commercial use and undergoing intellectual property protection. Details of the training and validation of the algorithm are available here: https://data.mendeley.com/datasets/hj9hmywh9c/1

RESOURCE AVAILABILITY

Lead contact
Andrew Beggs (a.beggs@bham.ac.uk).

Materials availability
Further information and requests for resources and reagents should be directed to and will be fulfilled by the lead contact, Andrew Beggs (a.beggs@bham.ac.uk).

Data and code availability
Images used in this study consist of potentially personally identifiable information and are available on completion of a signed data agreement with the Department of Health and Social Care via the lead contact. The algorithm itself is current undergoing intellectual property protection and on completion of this further details can be released on reasonable request. Any additional information required to reanalyze the data reported in this paper is available from the lead contact upon request. Details of algorithmic training are available here: Mendeley Data: https://data.mendeley.com/datasets/hj9hmywh9c/1 (key resources table).

EXPERIMENTAL MODEL AND SUBJECT DETAILS

Algorithm details
The algorithm was trained (details of training methodology available at: https://data.mendeley.com/datasets/hj9hmywh9c/1) on a set of photos from an artificially constructed set of LFDs that were hybridised with a known viral load of X-ray inactivated SARS-CoV-2 (England strain 1), at viral loads of 10000,1000,100,10 and 1 PFU/mL diluted at a 1:1 ratio of virus to LFD manufacturer (Innova) supplied dilution buffer (Ct values shown in Table S1). The LFDs consisted of a variety of cartridge shapes and these LFDs were then photographed using several devices (iPhones, Android phones) across a range of photographic conditions (light, dark, blurred, angled < 30 degrees, with plain and newsprint backgrounds) using standard device photographic settings to simulate the real world. Further validation and training of the algorithm was then undertaken using a set of LFD photos that were linked using a standardised app to RT-qPCR swab results taken at the same time as the LFD swab and underwent RT-qPCR using the ThermoFisher TaqPath SARS-CoV-2 kit.[4]

The AI LFD read uses a cascade of machine learning and image analysis methods to read the LFD and provide a quality control of the input. The algorithm was implemented in PyTorch and consists of networks which localise the region of interest; detect objects of

CellPress
OPEN ACCESS

interest; classify the objects of interest, using a series of subnetworks. In total there are 68 layers in the model. The LFD read algorithm uses a deep learning approach with a convolutional and multiscale network to identify the LFD read area and then interpret the control and test lines within the read area. The algorithm classifies the test as one of three categories: *positive* when the two lines are present; *negative* when only a control line is present; and *void* when no control line is present. This classification includes a score output by the final sigmoid layer of the network (range 0–1) which we call the ''confidence score''. The approach also includes a number of quality control (QC) measures aimed to reduce misclassifications from poorly acquired photos, for instance photos which are dark, blurry, or where the test in the image is extremely small. In addition to these QC measures, a deep autoencoder network is trained to learn a representation of a normal test. The more a new test deviates in appearance from the training examples (blur, lighting, pen markings, damage and decolouration of the test) the higher the anomaly score. These QC measures were reinforced for false positives, due to the skew between the number of positive and negative cases in real world testing scenarios. These mitigations include the use of a General Adversarial Network for identification of anomalies in the test read area (using the premise that, when presented with an image considerably different than that which it was trained upon, it will be unable to reconstruct the algorithm properly), further image quality measures and a minimum confidence threshold for the AI algorithm's classifications. These algorithms were also implemented in Pytorch. When one or more QC measures are activated, an inconclusive result is returned to the user and in production the user will be requested to retake the photo. Together, the AI classification algorithm and QC measures form the core of the MagnifEye^{TM} software tool. Deployment in production is based on an Azure architecture using Kubernetes with NVIDIA Tesla V100 GPUs.

In both sub-studies, MagnifEye's interpretation (positive, negative, void or inconclusive) was then passed to a results database where it was logged alongside the test subject's own asserted result. When a void result was observed (no control line, showing the test had failed), the study participant was instructed to take another test with a new test kit. Study subjects then were asked to take a picture of the void test before proceeding with a new test.

The AI interpreted result was not returned to the user during either sub-study, but instead analysed and kept as evidence to assess the accuracy of the algorithm reading against the reported outcome by the lay user (sub study 2) and trained operator (sub study 1).

The current quality control (QC) steps lead to an automatic rejection of 18.5% of cases. Examples of reasons for automated rejection include incorrect test device, discolouration of the test read area, plastic covering the test, rotation and incorrect scale, or shadowing on the test read area interfering with the read (Figure S1). In the 18.5% of the cases for which the QC algorithms reject the type of images shown above, the user is asked to retake the photo to have a chance of improving its quality and hence its likelihood of not being rejected by the QC algorithms.

Participants

The study was undertaken within Asymptomatic Testing Sites (ATSs, Sub study 1) and in NHS primary care staff, and Adult Social Care staff, visiting professionals and visitors (sub study 2) throughout the UK. ATS were swabbing centres based in the community used for testing of community participants for COVID-19. The sites chosen for this study did not overlap with those used to train the model.

Participants were included in the study if they were:

- Willing to participate in the study
- Aged 18 years or above *or* adolescents aged 12–17 (self-test and report with adult supervision) *or* children under 12 (should be tested and reported by an adult)
- Without any common COVID-19 symptoms
- Able (in the Investigators' opinion) and willing to comply with all study requirements

Participants were excluded from the study if they did not agree with privacy statement, had any common COVID-19 symptoms or any other significant disease or disorder which, in the opinion of the Investigator, may either put the participants at risk because of participation in the study, or may influence the result of the study, or the participant's ability to participate in the study.

Recruitment occurred between 12th - 31st March 2021. A total of 59,164 images were returned for sub-study 1 and 58,667 images for sub-study 2. For sub-studies 1 and 2, user age ranges across all cohorts were 16–80 years, representing all gender identities. For Sub-study 2, demographic data for 40,108 (n = 50,999) users was reported. Users aged 41 to 50 years old accounted for 22.67% of cases, followed by 51–60 (20.45%), and 31–40 (17.99%). The over 60s accounted for less than 6% of users, and under 30s for just under 12%. Very few voids were reported and the age distribution of the users submitting them was not statistically significant; however, when looking at the distribution of invalid images (I.e., images rejected by MagnifEye for not meeting one or more quality control measures) overall, 7.37% of images submitted were rejected. The images submitted by under 30s are statistically ($p < 0.001$) less likely to be rejected (6.0%), and the proportion increases with age at 6.18% for 31–40s, 6.77% for 41–50s, 8.86% for 51–60s, 11.30% for 61–70s, and 14.94% for over 70s.

Users that identified as female made up 67.70% (31,508) of all cases, while 10.95% (5,584) identified as male, with the remainder not declaring a gender. Male users were slightly more likely to submit invalid images (8.33%) than female users (7.28%). Where ethnicity was declared, users ranged across 23 different ethnicities. No statistically significant differences have been identified across different ethnicity groupings. Similarly, no significant differences were detected across use cases. Geographic spread was

largely dependent on the number of Asymptomatic Test Sites, NHS trusts and Care Homes that participate in the study and were representative of all United Kingdom regions. Although a sub study on usability was carried out, it did not specifically aim to understand the utility of the reader for assisting those who may have difficulty interpreting the result as so is not reported here.

METHOD DETAILS

Study design

Data collection was conducted according to a pre-planned protocol before the reference and index tests were performed. The reference test was defined as a visual inspection of an LFD by a trained member of staff within an asymptomatic testing site (ATS) or as a self-read by a participant. The index test was defined as the resulting photo of the lateral flow device taken either by the self-test participant or ATS user, that was uploaded to a web-based portal to be read by the machine learning algorithm.

This observational prospective study consisted of two sub studies, the first (Sub study 1) being a diagnostic performance evaluation study and the second (Sub study 2) being both a diagnostic performance evaluation study and also a human factors and usability performance study. Both were carried out in March 2021 and participants were recruited as a convenience series.

Ethical approval was sought from the Public Health England Research Ethics committee, who confirmed that the study was exempt from ethical approval, as the study was purely observational with the AI result not being returned to the user.

Image capture

Sub-study 1: Images were captured at Asymptomatic Test Sites (ATSs). A COVID-19 antigen test using Innova LFD was performed as per manufacturer's IFU, then the trained operative supervising the LFD read the test results, took a photo of the completed test using a standardised device and entered/uploaded their interpretation of the result using an NHSD web service as per written instructions. The pictures of completed tests were taken at the point at which the result was interpreted/decided.

Sub-study 2: Participants were invited to take images on their own mobile devices (such as iOS or Android smartphone or internet-enabled tablet) and upload these images using the NHSD web service. Study participants self-tested themselves using an Innova LFD as per manufacturer's instructions for use (IFU). Pictures of completed tests were taken at the point at which the result is interpreted as per manufacturer's IFU. This was tested in user research and participants were advised that they should take the test, perform another activity for 30 minutes (as per the manufacturers IFU) and then come back to take their photograph. At this point the user entered their interpretation of the result.

Index test (AI read) results were then compared with reference standard (ATS operator's read) results. The result recorded at the ATS by the trained operative was regarded as the 'ground truth' against which the AI result can be compared in the analysis phase. All positives and voids (ATS read or AI read), all discrepancies (where there was conflict between ATS and AI read excluding invalids, i.e. photos that did not meet the minimum photo criteria), and a sample of negatives were then reviewed by an independent clinical team providing further readings of the images whilst being unaware of the reason they were providing this read. These additional readings were referred for statistical analysis and comprised the final 'ground truth' for comparison against the AI read. The same process was carried out for self-reported images.

Resolution of discrepancies & intra-observer reliability

During this study, data was collected from three (B, C and D) different variations (cartridge shape) of Innova devices. In addition to the manually read and AI generated results, a third-party independent inspection by a trained expert clinician was performed for a subset of images to create a 'most trusted' result against which the sensitivity and specificity of the AI read can be measured to demonstrate the effectiveness of the AI algorithm, showing that it is 'at least as good as the average user read'. This was performed for both sub-studies.

The individuals chosen to undertake the assessment were three medical doctors with at least 5 years post qualification experience of working in a GCP compliant laboratory environment and interpreting test data. Initially, these independent readers underwent a qualification process. These doctors were trained by being provided with a sample of 50 images of laboratory produced LFD results where viral load was standardised for each LFD, and acted as ground truth. The results of the training images were known as these had been created by the Chief Investigator for the purposes of training the AI algorithm to recognise positive samples at differing strengths. The images selected were both positive and negative, plus a sample of voided cassettes. These were then blind read and the results returned to a project manager who compared each reading against the known result. Readers were only signed off as suitable if they correctly matched all images with the ground truth. If the reviewers' agreement was absolute (3 out of 3), we took their interpretation to be the "ground truth". In marginal cases where the reviewers disagreed fully or only had 2 out of 3 votes in cases where a positive could be present, a fourth reviewer was recruited (the Chief Investigator) to make a final decision.

The samples reviewed by the independent inspectors fell into 3 categories:

- Firstly, all samples where there was a discrepancy between the AI result and the result reported by the test subjects;
- Secondly, all samples where either the AI algorithm or the test subject returned a positive or void (but not where MagnifEye returned an inconclusive, defined as when the photo failed to meet one or more of the predefined QC measures)

- Thirdly, a random sample (1% of total) of images of negative results where there was consensus between the human and the AI algorithm). For Sub-Study 1 data, the sample of negatives was taken across the whole set independent of submission data (n = 568). For Sub-Study 2, 1% of the daily submissions with negative agreement were reviewed (n = 376).

Assessment took place via interpretation of the submitted image of the test subject's LFD. Each inspector individually assessed the image and recorded their interpretation of the result against the unique image identifier. The individual assessments were subsequently compared to determine a consensus amongst the inspectors and a final 'third party review' data point was recorded against the unique image identifier. A majority decision (two out of three reviewers concurred) view was taken as "ground truth". We chose this metric as ground truth rather than an orthogonal test such as real time PCR, as the primary outcome of this study was to understand the accuracy of the reader, rather to ascertain the accuracy of LFD against PCR, which have been shown to have different dynamic ranges of detection.

QUANTIFICATION AND STATISTICAL ANALYSIS

Sample size

Prevalence of the SARS-CoV-2 virus at the time of this study (March 2021) was low. We calculated that we would need at least 50k samples (based on 0.2% prevalence of SARS-CoV-2 infection) before we could observe a reasonable number of positive samples (at least 100) in order to produce a good estimate of the algorithm's sensitivity. Based on the initial training and internal validation of the algorithm, observed sensitivity and specificity were expected to be 95% or above during external validation with at most 1% error. For sub-study 1, the dataset extracted contained 56,776 valid images with 93 reported positives and later 126 confirmed positives which we found to be adequate to provide an accurate estimate of the relevant quantities.

Had the number of positives in the initial sample been too small to result in a useful estimate of the confidence interval of sensitivity, a Bayesian sequential sampling approach had been planned using a Rao-Blackwell approach to produce an unbiased estimator.[14,15]

The initial focus was on estimating the Area Under the Receiver Operating Characteristic Curve (AUROC). Since specificity was likely to be high, we aimed for an operating point on the AUROC to be sufficiently sensitive to positives to ensure a minimum sensitivity of 95%. We used bootstrap to assess the variation of the AUROC and derive confidence intervals for the metric but also for specificity and sensitivity. Once the confidence intervals for these measures were small enough or show signs of convergence, we reassessed the study and determined that no further sampling was necessary. We also observed and noted the Brier score for this model. If we noted substantial deviation from what is considered appropriate (0 to 0.25), we reassessed the model for overfitting and tried to identify other deviations that could have led to issues with discrimination. This tool was used for monitoring purposes only. In order to assess cross-entropy loss, we used the log loss approach to monitor the discrepancy between the positive or negative label (from the trained reviewers) and the algorithm result. We did not expect the model to be perfect (log-loss of 0), but we used this approach to identify when and if the model was possibly confidently wrong and used key findings to issue recommendations to improve quality control.

Calculation of accuracy

Diagnostic accuracy assessment was assessed with the standard metrics. We defined the index test as an automated analysis of clinical data by the AI-based MagnifEye software, and analysis of trained operator test results was used as a reference test. Relevant metrics with reliability within 95% of the confidence interval were calculated. We pre-specified a minimum sensitivity of greater than 95% and a specificity as close as possible to 100% for the AI algorithm classification to be deemed satisfactory.

ADDITIONAL RESOURCES

Study registration

This study is registered with the ISRCTN registry (reference number ISRCTN30075312) and further details can be found here: https://www.isrctn.com/ISRCTN30075312?q=ISRCTN30075312.

Cell Reports Medicine, Volume *3*

Supplemental information

Machine learning for determining lateral flow device results for testing of SARS-CoV-2 infection in asymptomatic populations

The LFD AI Consortium

Supplementary Materials for Beggs et al

Supplementary table 1: Table of viral loads in reference specimens as determined by real time PCR of ORF1ab and N genes vs. supplied concentrations of PFU/ml. Related to Figure 2.

PFU/ml	Ct ORF1ab	Ct N
10000	17.9	21.7
1000	24.3	27.4
100	27.4	30.3
10	33.5	35.2
1	35.6	38.6

List of affiliations of secondary authors:

NHS Test & Trace: Robert Banathy, Mark Branigan, Paul Lewis-Borman, Nishali Patel, Lennard Lee, Tom Fowler

Durham University; Camila C. S. Caiado

Sensyne Plc: Anna Dijkstra, Piotr Chudzik, Paria Yousefi, Avelino Javer, Bram Van Meurs, Lionel Tarassenko, Benjamin Irving

University of Birmingham: Celina Whalley, Neeraj Lal, Helen Robbins, Elaine Leung, Andrew D Beggs
